# Inter-observer agreement of canine and feline paroxysmal event semiology and classification by veterinary neurology specialists and non-specialists

**DOI:** 10.1186/s12917-015-0356-2

**Published:** 2015-02-18

**Authors:** Rowena MA Packer, Mette Berendt, Sofie Bhatti, Marios Charalambous, Sigitas Cizinauskas, Luisa De Risio, Robyn Farquhar, Rachel Hampel, Myfanwy Hill, Paul JJ Mandigers, Akos Pakozdy, Stephanie M Preston, Clare Rusbridge, Veronika M Stein, Fran Taylor-Brown, Andrea Tipold, Holger A Volk

**Affiliations:** Department of Clinical Science and Services, Royal Veterinary College, Hertfordshire, UK; Department of Veterinary Clinical and Animal Sciences, Faculty of Health and Medical Sciences, University of Copenhagen, Frederiksberg, Denmark; Department of Small Animal Medicine and Clinical Biology, Faculty of Veterinary Medicine, Ghent University, Ghent, Belgium; Cornell University College of Veterinary Medicine, Ithaca, New York USA; The Referral Animal Neurology Hospital “Aisti”, Vantaa, Finland; Animal Health Trust, Lanwades Park, Newmarket, UK; Fernside Veterinary Centre, Hertfordshire, UK; Department of Clinical Sciences of Companion Animals, Utrecht University, Utrecht, The Netherlands; University Clinic for Small Animals, Clinical Department for Companion Animals and Horses, University of Veterinary Medicine, Vienna, Austria; School of Veterinary Medicine, Faculty of Health & Medical Sciences, University of Surrey, Surrey, UK; Department of Small Animal Medicine and Surgery, University of Veterinary Medicine Hannover, Buenteweg 9, 30559 Hannover, Germany

**Keywords:** Seizure, Epilepsy, Canine, Feline, Video, Paroxysmal event, Agreement, Kappa

## Abstract

**Background:**

Advances in mobile technology mean vets are now commonly presented with videos of paroxysmal events by clients, but the consistency of the interpretation of these videos has not been investigated. The objective of this study was to investigate the level of agreement between vets (both neurology specialists and non-specialists) on the description and classification of videos depicting paroxysmal events, without knowing any results of diagnostic workup. An online questionnaire study was conducted, where participants watched 100 videos of dogs and cats exhibiting paroxysmal events and answered questions regarding: epileptic seizure presence (yes/no), seizure type, consciousness status, and the presence of motor, autonomic and neurobehavioural signs. Agreement statistics (percentage agreement and kappa) calculated for each variable, with prevalence indices calculated to aid their interpretation.

**Results:**

Only a fair level of agreement (*κ* = 0.40) was found for epileptic seizure presence. Overall agreement of seizure type was moderate (*κ* = 0.44), with primary generalised seizures showing the highest level of agreement (*κ* = 0.60), and focal the lowest (*κ* =0.31). Fair agreement was found for consciousness status and the presence of autonomic signs (κ = 0.21–0.40), but poor agreement for neurobehavioral signs (κ = 0.16). Agreement for motor signs ranged from poor (κ = ≤ 0.20) to moderate (κ = 0.41–0.60). Differences between specialists and non-specialists were identified.

**Conclusions:**

The relatively low levels of agreement described here highlight the need for further discussions between neurology experts regarding classifying and describing epileptic seizures, and additional training of non-specialists to facilitate accurate diagnosis. There is a need for diagnostic tools (e.g. electroencephalogram) able to differentiate between epileptic and non-epileptic paroxysms.

**Electronic supplementary material:**

The online version of this article (doi:10.1186/s12917-015-0356-2) contains supplementary material, which is available to authorized users.

## Background

Diagnosing and appropriately treating canine epilepsy requires accurate epileptic seizure detection and description of seizure semiology, the detailed observations of physical signs during a seizure episode indicative of an alteration in neurological state. Seizure semiology is a simple and cost-effective tool in the understanding of a seizure disorder and attempting to localise the epileptic focus. As such, veterinary neurology specialists and first opinion practitioners require a detailed semiologic description of paroxysmal events to confidently diagnose canine epilepsy and categorise the type of seizures experienced. Semiologic descriptions are often obtained from the family or caregivers of human epilepsy patients, relying on common terms for ictal symptoms [1]; however, video-EEG (electroencephalogram) monitoring is the preferred method for the diagnosis and classification of seizures due to its increased reliability [2]. In veterinary medicine such methods are not widely available, and have doubtful reliability, and thus it is of high importance to establish the accuracy and consistency of observational reports. These reports may come from the owners of affected dogs; however, due to the acute-onset, unpredictable and highly stressful nature of a seizure event, the reliability of these reports may be significantly reduced. Many owners now have access to mobile technology such as smart phones and tablet computers with video-recording capabilities, facilitating the recording of these events, which can later be presented to their veterinary surgeon. This was demonstrated in a recent study of the video-sharing website YouTube, where many owners uploaded videos of their dog’s seizure activity either seeking advice from viewers (2/3^rd^) or to show to their veterinarian (1/3^rd^) [3]. The consistency of the interpretation of these videos by different vets is therefore an area of importance. If agreement between vets in the classification of videos of paroxysmal events is low, then vets should ensure that videos are not used in isolation of other clinical data such as signalment, history and other diagnostics to diagnose epileptic seizures.

### Aims

This study aimed to investigate the level of agreement between vets (both recognised neurology specialists and non-specialists) on the description of videos depicting paroxysmal events without knowing the results of diagnostic workup. As the aim was limited to evaluating the phenotype of the event only, the observers were blinded to any additional history, diagnostics or treatment outcome for all animals. Finding good agreement between observers allows judgements to be made by different observers with some confidence in their consistency, whereas finding poor agreement between observers can highlight deficiencies in classification systems, which may indicate a need for refinement of definitions, or improved training of observers. The level of agreement between veterinary observers has important practical implications, for example, high agreement between observers is essential in multicentre clinical trials. Thus, this study aimed to highlight areas where further discussion is required, to improve consistency between neurologists diagnosing seizure disorders.

The focus of this study was on the initial perception of whether a paroxysmal event was a seizure or not, and if so, what type of seizure was present. As seizure type is likely predicted by the semiology of the event, the level of agreement between observers over (i) the quality of consciousness in the patient, in addition to (ii) the presence of 13 motor signs, (iii) three autonomic signs and (iv) three neurobehavioural signs was investigated. As this was a novel study, the aspects of seizure semiology investigated were intentionally broad as not to exclude potentially useful characteristics, that if demonstrated to show high concordance between observers could be useful predictors of seizure type.

In addition this study sought to detect differences in the reporting of seizure semiology and classification of seizure type between specialists and non-specialists, to investigate whether there is an effect of additional training upon semiologic description. Finally, this study sought to identify which observer-perceived seizure characteristics predict reported seizure type. If the characteristics used by observers to predict certain seizure types are not highly agreed upon then this could lead to unreliable classification of seizure type.

## Hypotheses

1. There are high levels of agreement between veterinary observers for the prediction of seizure presence and seizure type.
2. There are high levels of agreement between veterinary observers for the classification of (i) consciousness status, and the presence of (ii) motor signs, (iii) autonomic signs and (iv) neurobehavioural signs.
3. There are differences in the classification of seizure presence and type, and reporting of seizure semiology between veterinary neurology specialists and non-specialists.
4. Observer-reported seizure semiology will differentiate between primary generalised seizures, focal seizures, and focal seizures with secondary generalisation.

## Methods

A questionnaire was hosted on SurveyMonkey© in April 2014, with 10 senior neurology specialists (9 Diplomates of the European College of Veterinary Neurology (ECVN) and 1 associate ECVN member), and 5 Royal College of Veterinary Surgeon (RCVS) registered non-specialist veterinary surgeons invited to participate. One hundred videos of dogs and cats (sourced: 15 videos from one of the authors and 85 uploaded to YouTube©) exhibiting paroxysmal events were provided. Videos that were initially sourced from YouTube© were identified using the search term “dog seizure” or “cat seizure”. All videos on YouTube are constantly shuffled by the website’s confidential search algorithms. To randomise video selection, every 5th video was selected for analysis; however, the following exclusion criteria were applied: only one video per uploader, very poor quality videos, professional videos, advertisements, photographic collages, videos not showing domestic dogs or cats, and videos with text overlying the footage. The resultant 85 YouTube©-sourced videos and 15 author-sourced videos were provided to participants at the start of the study.

Participants were requested to watch all 100 videos and initially answer the following questions:

1. Is what you see in the video an epileptic seizure?
2. If you think it is NOT an epileptic seizure, what term would you use to describe the episode?

Participants were allowed to view the videos as often as they wished and were allowed to review the video as they were answering the survey. If the participant responded that they believed it was *not* an epileptic seizure they were instructed to move on to the next video. If participants responded that the video did indeed show an epileptic seizure, they were requested to categorise what the seizure type was best classified as (focal, focal with secondary generalisation or primary generalised). Participants were then requested to further characterise the epileptic seizure based on the presence and type of motor signs, autonomic signs and neurobehavioural signs and quality/status of consciousness, which were listed as tick boxes with the option to select as many as believed to apply (Additional file 1). This study was approved by the Royal Veterinary College Ethics and Welfare Committee.

### Statistical analysis

#### Hypothesis 1 and 2: are there high levels of agreement between veterinary observers for the prediction of seizure presence and type, the classification of consciousness status, and the presence of motor, autonomic and neurobehavioural signs?

Agreement statistics were calculated using Minitab version 17. Raw percentage (%) agreement, the percentage of the total number of observations for each variable where there is agreement was calculated for each variable across all videos, with the mean and 95% CI reported for each variable. Percentage agreement should not be solely relied upon however, as it does not take into account chance agreement, and thus to be more stringent, Fleiss’ kappa (κ) for more than 2 observers was calculated for each variable in the questionnaire to determine which aspects of a seizure were agreed upon between observers [4].

This study compared observers equally against one another, rather than against an objective method or a trained individual and thus no ‘gold standard’ was used to compare ratings with. Good agreement was indicated by % agreements close to 100 and by *κ* values close to 1. In line with Benbir et al. (2013), concordance was rated as ‘poor’ for κ values ≤ 0.2; ‘fair’ if κ were in the range 0.21–0.40; ‘moderate’ for 0.41–0.60; ‘good’ for 0.61–0.80; and ‘excellent’ if κ exceeded 0.81. This was an exploratory study to see which aspects of a seizure were most or least agreed upon, and thus a minimum threshold of *κ* was not set. As a guide, the minimum threshold for κ is often arbitrarily set at κ ≤ 0.4 [5].

**Table 1:** **Inter-observer agreement of seizure presence, type and consciousness status**

**Variable**	**Mean % agreement (95% CI)**	**PI**	**Category**	**κ**	**SE (κ)**	**Z**	***p***
**Is this an epileptic seizure?**	29% (19-40%)	0.4	Yes	0.40	0.01	35.12	<0.001
**What type of epileptic seizure?**	18% (10-28%)	0.47	Focal seizure	0.31	0.01	27.36	<0.001
		0.86	Focal seizure with secondary generalisation	0.53	0.01	45.63	<0.001
		0.27	Primary generalised seizure	0.60	0.01	51.93	<0.001
			Overall	0.44	0.01	62.08	<0.001
**Is the dog conscious?**	9% (3-19%)	0.72	Conscious (No impairment)	0.45	0.01	39.92	<0.001
		0.38	Impairment in consciousness	0.20	0.01	15.16	<0.001
		0.48	Unconscious	0.54	0.01	41.99	<0.001
			Overall	0.39	0.01	42.59	<0.001

(κ = kappa, PI = prevalence index, SE = standard error).

**Table 2:** **Inter-observer agreements of motor signs present in epileptic seizures**

**Variable**	**Mean % agreement (95% CI)**	**PI**	**κ**	**SE (κ)**	**Z**	***p***
**Does the dog show motor signs?**	73% (63-83%)	0.92	0.06	0.01	4.84	<0.001
Were movements more present on the left?	77% (67-86%)	0.88	0.28	0.01	24.59	<0.001
Were movements more present on the right?	67% (56-77%)	0.86	0.07	0.01	6.05	<0.001
Was the head turned to the side?	63% (51-73%)	0.84	0.14	0.01	11.95	<0.001
Were there running movements?	59% (48-70%)	0.40	0.58	0.01	50.27	<0.001
Were there rhythmic jerks around the mouth?	46% (35-57%)	0.40	0.44	0.01	38.35	<0.001
Was there rhythmic pelvic limb movements?	39% (28-50%)	0.12	0.56	0.01	48.79	<0.001
Are there rhythmic thoracic limb movements?	35% (25-46%)	0.17	0.55	0.01	48.02	<0.001
Was there oral movement (lip smacking)?	34% (24-45%)	0.20	0.35	0.01	30.43	<0.001
Was there stiffening of the pelvic limbs?	28% (19-39%)	0.42	0.37	0.01	33.80	<0.001
Was there stiffening of the thoracic limbs?	25% (16-36%)	0.01	0.39	0.01	34.09	<0.001
Were movements equal between both sides?	18% (10-28%)	0.04	0.37	0.01	32.00	<0.001
Are the eyes open?	5% (1-12%)	0.16	0.17	0.01	15.13	<0.001

(κ = kappa, PI = prevalence index, SE = standard error).

**Table 3:** **Inter-observer agreement of autonomic and neurobehavioural signs in epileptic seizures**

**Variable**	**Mean % agreement (95% CI)**	**PI**	**κ**	**SE (κ)**	**Z**	***p***
**Does the dog show autonomic signs?**	10% (4-18%)	0.09	0.28	0.01	24.35	<0.001
Did the dog salivate?	53% (42-64%)	0.51	0.64	0.01	55.24	<0.001
Did the dog urinate?	92% (83-97%)	0.97	0.17	0.01	14.89	<0.001
Did the dog defecate?	99% (93-100%)	0.99	−0.01	0.01	−0.07	0.5298
**Does the dog show neurobehavioral signs?**	4% (1-10%)	0.15	0.16	0.01	14.09	<0.001
Did the dog show fear/anxiety?	22% (13-32%)	0.61	0.15	0.01	12.66	<0.001
Did the dog show aggression?	86% (76-92%)	0.96	0.17	0.01	14.48	<0.001
Did the dog appear to hallucinate?	65% (54-75%)	0.89	0.13	0.01	11.48	<0.001

(κ = kappa, , PI = prevalence index, SE = standard error).

A limitation of the kappa statistics is that the magnitude of *κ* is a function of the prevalence of the trait measured by a question as well as the number of discordant responses [6,7] and thus a skewed distribution of data lowers the *κ* coefficient. In near-homogenous populations, evidence for agreement above chance levels is difficult to identify, resulting in low *κ* values. To aid the interpretation of κ values and % agreement for each variable, the prevalence index (PI) for multiple observers [8] was calculated using the following formula:

$$ \mathrm{PI}=\frac{{\mathrm{R}}_1\hbox{-} {\mathrm{R}}_2}{N_n} $$

PI = The absolute difference between the number of ratings in categories 1 and 2 (*R*_1_ and *R*_2_, respectively), divided by the number of subjects (*N*) multiplied by the number of ratings per subject (*n*).

Where high κ and % agreement values are achieved (and are of a similar magnitude), PI may not need to be consulted; however, if κ is low, or if the κ and % agreement values disagree, the PI can aid in the interpretation of this result. For example, if the % agreement is high but κ is low, this result is inconclusive due to the PI being too high, rather than due to clear inconsistency between observers. Where κ is low, but % agreement is correspondingly low, this is due to inconsistency between observers, and the variable should be considered unreliable. Such occurrences are highlighted in the results.

#### Hypothesis 3: are there differences in the classification of seizure presence and type, and reporting of seizure semiology between veterinary neurology specialists and non-specialists?

The influence of experience and training was investigated by analysing associations between observer type (specialist *vs*. non-specialist) and seizure type/characteristics, using IBM SPSS v19. The binomial dependent variable was neurology specialist or non-specialist, and the independent variables were seizure presence, type, consciousness status and the presence of motor (13), autonomic (3) and behavioural (3) signs. Associations were screened at the univariate level using Chi-squared analysis for categorical variables. If significant variables were identified they were taken forwards to a binary mixed model, where video number and observer were included as random effects to control for these sources of non-independence.

#### Hypothesis 4: does observer-reported seizure semiology differentiate between primary generalised seizures, focal seizures, and focal seizures with secondary generalisation?

Multinomial mixed model analyses were carried out in IBM SPSS v19 to determine which factors influenced the choice of seizure type. Three dependent variables were used in the multinomial models: primary generalised seizures, focal seizures, and focal seizures with secondary generalisation. Independent variables in the model were the observer-perceived consciousness status, and presence of thirteen motor, three autonomic and three neurobehavioural signs. All independent factors were first tested at the univariable level using Chi-squared analysis to identify significant factors for inclusion in the multinomial model, with P < 0.2 considered for inclusion. A backward stepwise model building strategy was used, selecting models based on fit, as determined by the Akaike information criterion (AIC) statistic, significance of terms included (P ≤ 0.05 was considered significant), and maximisation of the correct percentage classification of cases. Multicollinearity was initially avoided via examining the associations between all nominal independent variables to detect any high levels of association. If found, the variable that resulted in better model fit was selected for the final model. All models were also checked for collinearity via inspection of the standard errors of the regression coefficients to see if they were inflated which would signify multicollinearity was a problem in that model.

## Results

To allow for statistical analysis, all videos must have been rated by an equal number of observers, and thus 17 of the 100 videos were excluded from the analysis due to missing data, and all ratings from 1 observer were excluded due to their low response rate to the questions. In total 1162 ratings were made of 83 videos by the remaining 14 independent observers.

### Hypothesis 1 and 2: are there high levels of agreement between veterinary observers for the prediction of seizure presence and type, the classification of consciousness status, and the presence of motor, autonomic and neurobehavioural signs?

#### Epileptic seizure presence and type

When questioned on whether the paroxysmal events in the videos represented epileptic seizures, 72% of responses to all videos reported they thought the event was a seizure; however, there was a fair level of agreement (*κ* = 0.40) with on average only 29% (95% CI 19-40%) agreement between observers as to whether it was a seizure or not for each video (Table 1). Overall agreement of seizure type was moderate (*κ* = 0.44), with on average only 18% agreement between observers across videos. The most common seizure type reported from the videos was primary generalised (36% of all ratings), with the highest level of agreement (*κ* = 0.60) of all types. The lowest level of agreement was for focal seizures (*κ* = 0.31).

#### Consciousness status

Very low% agreement was achieved regarding the consciousness status of the dog, with on average 9% agreement between observers as to whether the dog was conscious during the paroxysmal events (Table 1). The poorest agreement was achieved for impairment in consciousness (*κ* = 0.20), versus moderate levels of agreement for unconscious (*κ* = 0.54).

#### Motor signs

When questioned on whether the paroxysmal events in the videos showed motor signs, 96% of responses to all videos reported they thought motor signs were present, with on average 73% agreement between observers as to whether motor signs were present (Table 2). As the PI was exceptionally high for this variable, with a homogenous sample dominated by ‘yes’ responses, the κ is artificially lowered to a level of poor agreement (κ = 0.06). The highest levels of agreement for individual signs, as determined by κ values, were whether there were running movements, whether there were rhythmic pelvic limbs movements and whether there were rhythmic thoracic limb movements (moderate agreement). The lowest levels of agreement as determined by κ values were for whether the eyes were open, whether the head was turned to the side and whether movements were more present on the right side. The latter two variables had high PIs and thus the sample population may be too homogenous to interpret these results.

#### Autonomic signs

When questioned on whether the paroxysmal events in the videos showed autonomic signs, 55% of responses to all videos reported they thought autonomic signs were present; however, % agreement was low with on average just 10% agreement between observers and a ‘fair’ κ value (κ = 0.28) (Table 3). There was good agreement as to whether the dog salivated in the video (κ = 0.64), but poor κ values for urination or defecation. There were high PIs for both urination and defecation owing to their rarity of reporting (1.4% and 0.1% of all ratings, respectively), and thus despite high % agreement (both with on average over 90% agreement between observers), their κ values were low and thus the reliability of these signs is inconclusive.

#### Neurobehavioural signs

When questioned on whether the paroxysmal events in the videos showed neurobehavioral signs, over half of responses reported that they were present (58%); however,% agreement was again very low with average agreement of just 4% across all videos and a poor κ value. κ values for all three neurobehavioural signs were poor; however% agreement was high for aggression and hallucination and thus their κ was artificially lowered due to the homogeneity of the sample and the rarity of their reporting (2% and 5% of all ratings, respectively). Fear and anxiety was reported in nearly a fifth of ratings (19%); however had both a poor κ and % agreement, thus indicating low levels of agreement of its presence.

#### Hypothesis 3: are there differences in the classification of seizure presence and type, and reporting of seizure semiology between veterinary neurology specialists and non-specialists?

Chi-squared analyses revealed significant differences in seizure semiology and classification between specialists and non-specialists. Specialists were less likely to report what they saw in the videos as a seizure than non-specialists (68% vs. 75%; p = 0.008). When questioned on what this was if *not* a seizure, specialists were more likely to report a movement disorder (53% vs. 43%; p = 0.047) and pain associated behaviour (3% vs. 0%; p = 0.047) than non-specialists. Specialists were less likely to report a seizure as focal (34% *vs*. 42%; p = 0.011), more likely to report impaired consciousness (47% *vs*. 37%; p = 0.003) and less likely to report unconsciousness (32% *vs*. 45%; p < 0.001) than non-specialists. With regard to motor signs, specialists were less likely to report the eyes as open (48% *vs*. 76%; p < 0.001), oral movement (36% *vs*. 47%; p = 0.001), rhythmic jerks around the mouth (28% *vs*. 34%; p = 0.031), stiffening of the thoracic limbs (46% *vs*. 56%; p = 0.003), rhythmic pelvic limb movements (41% *vs*. 50%; p = 0.005) or that movements were equal on each side (44% *vs*. 55%; p = 0.001) than non-specialists. There was no difference in the reporting of autonomic signs between specialists and non-specialists. The only difference in the reporting of neurobehavioural signs was that specialists were more likely to report aggression than non-specialists (5% *vs*. 1%; p < 0.001). There were differences in the perception of duration, with non-specialists less likely to report short episodes of only seconds (5% vs. 9%; p = 0.005) or less than 1 minute (27% vs. 45%; p < 0.001) than specialists. When a binary mixed model analysis was attempted to determine which factors were associated with the observer being a specialist or a non-specialist, no factors were found to be significantly associated when video number and observer were included as random effects.

#### Hypothesis 4: does observer-reported seizure semiology differentiate between primary generalised seizures, focal seizures, and focal seizures with secondary generalisation?

At the univariate level, Chi-squared analysis identified several factors associated with the classification of seizure type including the presence of motor (p < 0.001), autonomic (p < 0.001) and neurobehavioural (p = 0.009) signs. Multinomial mixed models identified seven factors significantly associated with reported seizure types: oral movement, stiffening of thoracic limbs, rhythmic thoracic limb movements, running movements, equal movements on each side, salivation, hallucination (Table 4 and Additional file 2: Table S1).

**Table 4:** **Multinomial mixed model analysis of which observer perceived seizure characteristics predict reported seizure type**

**Risk factor**		**Focal vs. primary generalised**				**Focal with secondary generalisation vs. primary generalised**				**Focal with secondary generalisation vs. focal**			
		**OR**	**95% CI**	**t**	***p***	**OR**	**95% CI**	**t**	***p***	**OR**	**95% CI**	**t**	***p***
Oral movement	No	0.45	0.25-0.81	−2.64	0.008	0.50	0.23-1.09	−1.74	0.082	0.98	0.44-2.20	−0.04	0.966
	Yes					*ref*							
Stiffening of thoracic limbs	No	7.82	4.39-13.96	6.98	<0.001	1.07	0.50-2.27	0.17	0.868	0.19	0.08-0.41	−4.21	<0.001
	Yes					*ref*							
Rhythmic thoracic limb movements	No	3.70	1.88-7.27	3.80	<0.001	1.85	0.73-4.68	1.31	0.191	0.64	0.24-1.65	−0.93	0.352
	Yes					*ref*							
Running movements	No	9.75	3.60-26.42	4.48	<0.001	1.49	0.58-3.85	0.82	0.409	0.17	0.05-0.56	−2.89	0.004
	Yes					*ref*							
Equal movements on each side	No	4.70	2.61-8.47	5.15	<0.001	2.37	1.07-5.27	2.13	0.034	0.78	0.34-1.79	−0.60	0.551
	Yes					*ref*							
Salivation	No	2.69	1.29-5.61	2.63	0.009	1.19	0.45-3.17	0.35	0.730	0.43	0.16-1.18	−1.64	0.102
	Yes					*ref*							
Hallucination	No	0.24	0.07-0.86	−2.19	0.029	0.37	0.07-2.08	−1.13	0.260	2.44	0.47-12.7	1.06	0.290
	Yes					*ref*							

(κ = kappa, SE = standard error, OR = odds ratio, *ref* = reference category).

Reports of oral movements were associated with classification as a focal seizure, with reports of their absence decreasing the likelihood of a report of a focal seizure 0.45 fold *vs.* a primary generalised seizure (p = 0.008). Reports of thoracic limb stiffening were associated with classification of primary generalised seizures, with their absence increasing the likelihood of classification as a focal seizure 7.83 fold *vs.* a primary generalised seizure (p < 0.001), and decreasing the likelihood of classification as a focal seizure with secondary generalisation 0.19 fold *vs.* a focal seizure (p < 0.001). Reports of rhythmic thoracic limb movements were also associated with classification of primary generalised seizures, with their absence increasing the likelihood of classification as a focal seizure 3.7 fold *vs.* a primary generalised seizure (p < 0.001). Reports of running movements were associated with classification as a primary generalised seizures and focal seizures with secondary generalisation, with their absence increasing the likelihood of classification as a focal seizure 9.75 fold *vs.* a primary generalised seizure (p < 0.001). In addition, the absence of running movements decreased the likelihood of classification as a focal seizure with secondary generalisation 0.17 fold vs. a focal seizure (p = 0.004). Reports of equal movements on each side of the body were associated with classification as primary generalised seizures, with reports of unequal movements increasing the likelihood of classification as a focal seizure 4.70 fold *vs.* a primary generalised seizure (p < 0.001) and increasing the likelihood of classification as a focal seizure with secondary generalisation 2.37 fold *vs.* primary generalised seizures (p = 0.034).

Reports of salivation were associated with the classification of primary generalised seizures, with reports of absence of salivation increasing the likelihood of classification as a focal seizure 2.69 fold *vs.* a primary generalised seizure (p = 0.009). Finally, reports of hallucination were associated with the classification of focal seizures, with reports of absence of hallucination decreasing the likelihood of classification as a focal seizure 0.24 fold *vs.* a primary generalised seizure (p = 0.029).

There was overlap in the prediction of seizure type for three aspects of seizure semiology, where their presence increased the likelihood of two seizure types. Stiffening of the thoracic limbs was associated with reports of a primary generalised seizure (rather than a focal seizure), but also reports of a focal seizure with secondary generalisation (rather than a focal seizure) (Table 4; Additional file 2: Table S1). Running movements were associated with reports of a primary generalised seizure (rather than a focal seizure), but also reports of a focal seizure (rather than a focal seizure with secondary generalisation). Finally, equal movements on each side of the body were associated with reports of a primary generalised seizure (rather than a focal seizure AND rather than a focal seizure with secondary generalisation).

## Discussion

### Hypothesis 1 and 2: are there high levels of agreement between veterinary observers for the prediction of seizure presence and type, the classification of consciousness status, and the presence of motor, autonomic and neurobehavioural signs?

Prior to this study, no data were available in the literature regarding inter-observer agreement for paroxysmal event semiology between vets. Contrary to our initial hypotheses of high levels of agreement between veterinary observers for the prediction of seizure presence, type and description of seizure semiology, this study has demonstrated that there was only fair-moderate inter-observer agreement in the description of seizure semiology between a cohort of veterinary neurology specialists and non-specialists as ascertained by κ analysis and percentage agreement, with prevalence indices to aid interpretation. No variables achieved excellent agreement, and the only variable to achieve good agreement was whether the dog salivated or not, followed by whether the seizure type was primary generalised, which nearly missed good agreement. Few of the variables showed poor agreement; however, neurobehavioural signs were the least agreed upon domain with consistently poor agreement ratings. There was on average only 29% agreement between observers as to whether a video represented a seizure event or not, achieving a κ value of just 0.4, a value that is commonly stated as the minimum threshold for reliability [5]. This suggests that in isolation, observing videos of paroxysmal events may be an unreliable way to diagnose a seizure, thus highlighting the importance of detailed history taking, physical examination and diagnostic testing in determining whether an epileptic seizure has occurred.

Similar studies have been carried out in human medicine, and parallels can be made with the results of this study [2]. Impairment of consciousness was the least agreed upon consciousness status category in this study, as has been demonstrated previously in a human epilepsy study [2]. That study also demonstrated that head turning was less well agreed upon than other variables in humans, which in this study only showed poor agreement. In comparison to that study, agreement between veterinarians is much lower than between human neurologists, for example concordance between two human neurologists was classed as good to excellent (using the same scale) for all 23 questions posed; however, it must be noted that in the human epilepsy study, raters were aware that the paroxysm was an epileptic seizure which may have improved agreement on aspects of semiology. Despite this, the relatively low agreement between veterinarians described by this study may justify further discussions between experts regarding semiologic descriptions, and further training to non-specialists, to improve levels of agreement.

One of the poorest areas of agreement was regarding the consciousness status of the dog, with particularly poor agreement for option ‘impairment in consciousness’. In the authors’ collective experiences, impairment in consciousness is often interpreted and reported by the observer when dogs are standing with a blank stare and apparently being incapable of recognizing owner/surroundings, for example they do not respond to commands, but it have been argued that impairment of consciousness cannot be objectively assessed in dogs [9]. Assessment of consciousness during epileptic seizures is generally not a simple topic; it is individually different, not always global, and “pieces” of consciousness (perception, cognition, responsiveness, memory function, motor performance) can be altered [10]. The oversimplification with categorization into conscious and unconscious was eliminated in the last human classification; however recognition of impairment remained an important point [1]. In animals, the responsiveness by motor function is the main (if not the only part) of consciousness which can be evaluated.

Classification of seizure type has implications for future multicentre treatment studies, as some medications used may have better effects on certain seizure types, so agreement here is of high importance. Focal seizures were the least agreed upon seizure type, which may be due to the complex array of signs that may be reported during them, including a variety of motor, postural, autonomic and behavioural signs [9]. One study has shown that neurobehavioral and autonomic signs are not uncommon in dogs and indicated that motor signs are not necessarily the most dominant clinical expression of a focal seizure [9]. Hallucination was thought to be associated with focal seizures in this study, a sign that may be potentially difficult to confidently and reliably recognise.

### Hypothesis 3: are there differences in the classification of seizure presence and type, and reporting of seizure semiology between veterinary neurology specialists and non-specialists?

As hypothesised, differences were seen in the classification of seizure presence and type, and reporting of seizure semiology between veterinary neurology specialists and non-specialists. These differences were limited to the univariate level, which may be due to the low sample size, particularly for non-specialists (n = 5). This has never been studied before and thus further study with a larger, balanced sample size of specialists and non-specialists may be warranted to confirm these results. At the univariate level, specialists were less likely to report what they saw in the videos as an epileptic seizure than non-specialists, which may be due to their experience of other, less common paroxysmal events (without seizure activity) that non-specialists may not recognise (e.g. specialists were more likely to report the paroxysmal event as a movement disorder than non-specialists such as idiopathic head bobbing, episodic falling, cramping syndrome), or may be more experienced in recognising more subtle signs (e.g. specialists were more likely to report pain associated behaviour than non-specialists) and thus categorising the episodes as non-seizure events. Reporting of motor, autonomic and neurobehavioral signs were similar between specialists and non-specialists, with the exception of several motor variables that non-specialists were more likely to report. It would be expected that the specialists would be more accurate than non-specialists owing to their training and experience, so it is possible that these were ‘false positives’ by the non-specialists rather than under recognition by the specialists. Specialists were more likely to report the presence of aggression than non-specialists, which may be due to the recognition of more subtle signs of aggression e.g. changes in body posture or facial expression rather than overt signs such as snarling or growling; however, as individual signs indicating aggression were not requested it is not possible to infer the cause of this difference. In a previous study of experienced and inexperienced people describing dog behaviour, observers showed little agreement when classifying aggression [11], so it is possible that additional training in this area from behavioural experts may be useful.

### Hypothesis 4: does observer-reported seizure semiology differentiate between primary generalised seizures, focal seizures, and focal seizures with secondary generalisation?

Only seven of the nineteen studied aspects of seizure semiology significantly differentiated between seizure type: oral movement, stiffening and rhythmic movement of the thoracic limbs, running movements, movements equally on both sides, salivation and hallucination. With regard to the five motor signs: oral movement, stiffening of the forelimbs and equal movements on each side only achieved fair kappa values, while rhythmic thoracic limb movements achieved moderate agreement and running movements almost reached good agreement, Salivation achieved a good level of agreement; however, hallucination had poor agreement. As observers use these aspects of semiology most prominently to differentiate between seizure type, emphasis should be made to train observers to recognise these characteristics to improve agreement for those with poorer levels of achievement. Factors that did not differentiate between any of the three seizure types may be considered less useful in the categorisation of seizure type in the future; however, due to this being a novel study, further data should be gathered before these criteria are discarded. When classifying seizure type, of the seven significant variables, those that are associated with only one seizure type may be considered most valuable, for example hallucination was only associated with focal seizures, in contrast to running movements which were associated with both primary generalised seizures and focal seizures with secondary generalisation, thus requiring further inquiry to differentiate.

### Statistical limitations

There were limitations to the statistics performed in this study, as in near-homogenous populations, evidence for agreement above chance levels is difficult to identify, resulting in low *κ* values [4]. Some variables in this study suffered from this problem, as reflected in very high prevalence indices (PI), meaning that the interpretation of their corresponding *κ* value was limited, with low *κ* values but high% agreement values reflecting inconclusive statistics rather than genuinely poor agreement. In future studies, to avoid this problem, a more balanced population should be initially selected; with approximately equal numbers of subjects in each category e.g. 50% of videos show seizures and 50% of videos show non-seizure paroxysmal episodes. To facilitate this, a gold standard observer would be required to determine the designation of each video; for example, a specialist not participating in the study, using videos of patients that had been diagnosed with epilepsy following full work up that had evidence of response to anticonvulsive treatment. Establishing an accepted gold standard may still be challenging due to the varying beliefs of neurology specialists. For example, some neurologists believe that Spike’s disease (Canine Epileptoid Cramping Syndrome) is a focal seizure, while others believe it is a movement disorder. Balancing the subjects in each category e.g. for the presence of each motor, neurobehavioral and autonomic sign may not be feasible for such a large sample.

Due to the presence of missing data, a direct comparison of agreement between the two sub-groups, specialists and non-specialists could not be carried out. This was due to agreement statistics requiring an equal amount of observers to rate an equal amount of videos, resulting in videos being removed from the analyses when missing data was present for that video. As different missing data was present, and thus different videos removed for specialists and non-specialists, this would not be a direct comparison. The alternative approach of analysing how these groups compared in their ratings was instead carried out.

### Video limitations

The variable quality of the videos used in this study due to their unstandardized online source may have influenced observers’ abilities to report on the features of the episodes. Recent studies have successfully used YouTube© videos to study neurological and behavioural problems in dogs [3,12], and its capacity to facilitate large-scale studies may counterbalance this limitation. This also reflects a real-life clinical situation where video quality is likely to vary between owners and between seizures e.g. those that happen in poor light conditions. A further limitation of using owner-recorded videos is that owners may not have video recording equipment to-hand when the seizure episode begins. This is particularly problematic when observers are differentiating between primary generalised and secondary generalised seizures, as missing the beginning of a secondary generalised seizure may lead observers to erroneously classify it as a primary generalised seizure.

### Limitations of experience categorisation

A further limitation of this study was the designation of ‘specialist’ vs. ‘non-specialist’, which may not capture the differences in experience between the veterinarians involved. All of the specialists had undergone extensive years of training in neurology; however, years as a specialist since this initial training was not considered, with additional years of experience potentially improving diagnostic accuracy. Some specialists may also have a clinical and/or research focus in epilepsy further increasing their experience. In addition, although classed as ‘non-specialists’, those veterinarians involved in this study had an interest in veterinary neurology and may have seen more relevant cases, making them different from other first opinion practitioners. As such, experience is more of a spectrum than a binomial trait. Whether the non-specialists were representative of all first opinion veterinarians or referral veterinarians of another specialism is also debatable as individual details were not requested here, and as such further study with a larger sample size may be needed to improve how representative these results are. A further limitation related to the observers was the amount of time participating veterinarians had available to view and rate the videos may have impacted upon their responses.

### Information required to supplement videos of paroxysmal events

An important limitation of our study (similarly in daily veterinary practice) is the lack of reliable method for the differentiation between epileptic and non-epileptic paroxysms. The current definition of epileptic seizure is “a transient occurrence of signs and/or symptoms due to abnormal excessive or synchronous neuronal activity in the brain” [13]. For a definitive diagnosis, the epileptic activity should be recorded, which is especially important in cases with unclear episodes. Advance in the veterinary EEG diagnostic is required. Video-EEG studies, more commonly used as a diagnostic tool in human neurology, could be used more widely in veterinary medicine to aid characterisation of episodes beyond what can be observed on a video alone. This additional information could potentially improve inter-observer agreement. A recent veterinary video-EEG study diagnosed a juvenile Chihuahua with subtle myoclonic absences with perioral myoclonia and head twitching [14]. The patient had been admitted for evaluation of what was suspected to be focal seizures, with a four-month history of recurrent episodes of head and nose twitching, associated with intermittent hind limb jerking and suspected staring for a duration of a few seconds. The author confirmed bilateral generalized synchronous 4 Hz spike-and-wave complexes on ictal EEG time locked with the episodes. The case represents the first confirmed absence seizure in dog. Without video-EEG the epileptic origin could only have been speculated [14].

In daily veterinary practice, some additional information is usually available that may be helpful for the assessment whether the paroxysmal event is of epileptic origin or not. This includes breed, age of onset, pre and postictal signs, precipitating event, duration of the event, occurrence of the event during daytime, laboratory results, neuroimaging findings and response to antiepileptic therapy. These data were not investigated (except breed) in the present study; however, it should be borne in mind that these results and the level of agreement could have been influenced by their inclusion.

### Further study

Further exploration of this area could include an inter-observer agreement study of seizure-episode videos between owners and neurologists to investigate the accuracy of reports they are provided with. In a study of human epilepsy [2], high concordance between physicians and caregivers was observed. This was not anticipated by the authors; with differences in training and experience expected to lead to reduced concordance. The authors speculated that because of long disease duration and high seizure frequency in the majority of patients, most caregivers are likely to have experienced several seizure episodes first-hand, and thus their increased familiarity with the condition would increase the similarity of their ratings with physicians. This could be investigated in veterinary patients, with owners with differing levels of experience of canine epilepsy (e.g. newly diagnosed vs. longer-term cases) and between owners of dogs experiencing different seizure phenotypes (e.g. high *vs*. low frequency, clustering etc.). If good concordance is seen between vets and owners, then greater confidence may be given to owner descriptions for those cases where videos are not provided.

## Conclusion

In conclusion, this study has demonstrated that there were relatively low levels of agreement of seizure presence, type and semiologies reported by veterinary neurology specialists and non-specialists, highlighting the need for ongoing debate regarding the descriptive terminology used for seizure semiology in veterinary medicine, and the need for further training in focussed areas. Although the use of videos to diagnose seizure activity may be increasingly common, the results presented here demonstrate that it should not be solely relied upon, with existing diagnostics always supplementing videos, and new diagnostics such as EEG more widely used for more objective, definitive diagnoses.

## Additional files

Additional file 1:
**Questionnaire hosted on SurveyMonkey® (repeated by each observer for 100 videos).**
Additional file 2: Table S1. Simplified schematic of significant associations between seven aspects of seizure semiology and observer-reported seizure type (adapted from Table 4). Yellow cells signify aspects of seizure semiology that were deemed to be associated with focal seizures, blue for primary generalised seizures, and red focal seizures with secondary generalisation.

## Abbreviations

ECVN: European College of Veterinary Neurology
RCVS: Royal College of Veterinary Surgeon
PI: Prevalence index
CI: Confidence interval
SE: Standard error
EEG: Electroencephalogram
