# Supplementary material for: Inter-observer agreement of canine and feline paroxysmal event semiology and classification by veterinary neurology specialists and non-specialists
Source: BMC Vet Res. 2015 Feb 18;11:39. doi: 10.1186/s12917-015-0356-2 (PMC4337258; doi:10.1186/s12917-015-0356-2)
Supplement: Additional file 1: — Questionnaire hosted on SurveyMonkey® (repeated by each observer for 100 videos). [file 12917_2015_356_MOESM1_ESM.docx]

**Additional file 1: Questionnaire hosted on SurveyMonkey® (repeated by each observer for 100 videos)**

**Q1: Observer name (select from list)**

**Q2: Video (Please add number; e.g. 12, 34 or 50....) _____**

**Q3: Is what you see in the video a seizure? (select one)**

- Yes
- No

**Q4: 4. If you think this is NOT a seizure, what term would you use to describe this episode?**

(free text) _________________________________________________________________________

**Q5: 5. If the video shows a seizure, please continue with the questionnaire. If the video does not show a seizure, please move on to the next video. You need to submit the online form by pressing "Done" at the end of the form before you can move on**

**This seizure can be best classified as? (select one)**

- Focal
- Focal with 2nd generalisation
- Generalised

**Q6. How would you classify the seizure (select one)**

- Myoclonic
- Dystonic
- Versiv
- Tonic
- Clonic
- Tonic-Clonic (seizure involving both components, in any order. Both can also occur simultaneously)
- Atonic

**Q7. Is the dog conscious during the episode? (select one)**

- Conscious (no impairment)
- Impairment in Consciousness
- Unconscious

**Q8. Are there any motor signs? If yes, please tick all correct answers**

- NO
- Eyes are open
- The head was turned to one side
- Manual automatic movement
- Oral movement like lip smacking
- Rhythmic jerks around the mouth
- Stiffening of the forelimb(s)
- Rhythmic forelimb(s) movement
- Stiffening of the hindlimb(s)
- Rhythmic hindlimb(s) movement
- Running Movement
- Movements are more present on the LEFT SIDE of the body
- Movements are more present on the RIGHT SIDE of the body
- Movements - BOTH SIDES are equally affected

**Q9. Autonomic signs present? If yes, please tick all correct answers**

- NO
- Salivation
- Urination
- Defecation

**Q10. Are there any neurobehavioral changes? If yes, tick all which apply**

- NO
- Fear/Anxiety
- Aggression
- Hallucination

**Q11: Any additional comments**

(free text) _________________________________________________________________________
