# Supplementary material for: Inter-observer agreement of canine and feline paroxysmal event semiology and classification by veterinary neurology specialists and non-specialists
Source: BMC Vet Res. 2015 Feb 18;11:39. doi: 10.1186/s12917-015-0356-2 (PMC4337258; doi:10.1186/s12917-015-0356-2)
Supplement: Additional file 2: Table S1. — Simplified schematic of significant associations between seven aspects of seizure semiology and observer-reported seizure type (adapted from Table 4). Yellow cells signify aspects of seizure semiology that were deemed to be associated with focal seizures, blue for primary generalised seizures, and red focal seizures with secondary generalisation. [file 12917_2015_356_MOESM2_ESM.docx]

Additional file 2: Table S1. Simplified schematic of significant associations between seven aspects of seizure semiology and observer-reported seizure type (adapted from Table 3). Yellow cells signify aspects of seizure semiology that were deemed to be associated with focal seizures, blue for primary generalised seizures, and red focal seizures with secondary generalisation.

| **Seizure semiology** | **Seizure type** | | |
| --- | --- | --- | --- |
|  | **Focal vs. Primary generalised** | **Focal with secondary generalisation vs. Primary generalised** | **Focal with secondary generalisation vs. Focal** |
| **If seizure characteristic is present** | | | |
| **Oral movement** | Focal more likely than primary generalised | *Does not differentiate* | *Does not differentiate* |
| **Stiffening of thoracic limbs** | Primary generalised more likely than focal | *Does not differentiate* | Focal with secondary generalisation more likely than focal |
| **Rhythmic thoracic limb movements** | Primary generalised more likely than focal | *Does not differentiate* | *Does not differentiate* |
| **Running movements** | Primary generalised more likely than focal | *Does not differentiate* | Focal with secondary generalisation less likely than focal |
| **Equal movements on each side** | Primary generalised more likely than focal | Primary generalised more likely than focal with secondary generalisation | *Does not differentiate* |
| **Salivation** | Primary generalised more likely than focal | *Does not differentiate* | *Does not differentiate* |
| **Hallucination** | Focal more likely than primary generalised | *Does not differentiate* | *Does not differentiate* |
